# Twin Pregnancies in Dairy Cattle: Observations in a Large Herd of Holstein-Friesian Dairy Cows

**DOI:** 10.3390/ani10112165

**Published:** 2020-11-20

**Authors:** Fernando López-Gatius, Irina Garcia-Ispierto, Ronald H. F. Hunter

**Affiliations:** 1 Transfer in Bovine Reproduction SLu, 22300 Barbastro, Spain; 2 Agrotecnio Centre, 25198 Lleida, Spain; irinag@ca.udl.cat; 3 Department of Animal Science, University of Lleida, 25198 Lleida, Spain; 4 Sidney Sussex College, University of Cambridge, Cambridge CB2 3HU, UK; vhladfield@btinternet.com; 5 Ladfield, Oxnam, Jedburgh TD8 6QZ, UK

**Keywords:** twinning, dead co-twin, early fetal loss, twin reduction, bilateral asymmetry, heat stress

## Abstract

**Simple Summary:**

Cows have two ovaries, two uterine horns, two oviducts, and a uterine body. Although they are commonly monovular, they can sustain twin or other multiple pregnancies to term. In cows with a single pregnancy, the embryo implants in the uterine horn that is on the side of ovulation where a corpus luteum develops. Multiple pregnancies are classified into bilateral (one or more embryos in each uterine horn) and unilateral (all embryos in the same uterine horn, right or left) and, in both cases, embryos occur on the side of their corresponding corpus luteum or corpora lutea. Multiple pregnancies are undesirable in dairy herds as they compromise the reproductive performance and productive lifespan of cows. The present study sought to: (a) gain information on the incidence of twin pregnancies, (b) assess and expand information on the relative incidence of bilateral twins compared to unilateral twins, (c) confirm corresponding laterality between embryos and corpora lutea, (d) tabulate the frequency of triplets or quadruplets, and (e) evaluate possible effects of environmental heat stress conditions on the incidence of multiple pregnancies. The data for this study were derived from the ultrasonographic examination of 1130 cows carrying twins, triplets, or quadruplets, and 3160 cows carrying singletons.

**Abstract:**

Multiple pregnancies have devastating consequences on the herd economy of dairy cattle. This observational study examines incidence patterns based on data from the ultrasonographic examination of 1130 multiple pregnancies in cows in their third lactation or more carrying twins (98.8%), triplets (1.1%), or quadruplets (0.08%), and 3160 of their peers carrying singletons. Cows became pregnant following a spontaneous estrus with no previous hormone treatments. Irrespective of a significant decrease (*p* < 0.0001) in the conception rate (28–34 days post-insemination) during the warm period of the year, the multiple pregnancy rate was similar for both warm (26.5%) and cool (26.3%) periods. The incidence of unilateral multiple pregnancies (all embryos in the same uterine horn) was higher than that of bilateral pregnancies (at least one embryo in each uterine horn): 54.4% versus 45.6% (*p* < 0.0001). This difference rose to 17% during the warm season (*p* = 0.03). Pregnancy was monitored in unilateral multiple pregnancies until abortion or parturition (*n* = 615). In the warm period, the parturition rate was 43% compared to 61% recorded in the cool period (*p* < 0.0001). Thus, a warm climate is the main factor compromising the fate of multiple pregnancies. Some clinical suggestions are provided.

## 1. Introduction

Low fertility and abortion are a major source of economic losses for dairy herds [1]. Pregnancy losses of a non-infectious nature mainly occur within the first 90 days of gestation [2,3,4], and parity (age) [5] and twin pregnancies [3,6] are the main non-infectious factors compromising pregnancy maintenance during this period. Recently, multiple ovulations and subsequent multiple pregnancy rates have increased in parallel with milk production. The economic burden of a twin pregnancy has been estimated at up to $225 [6]. Twin pregnancies are classified into bilateral (one fetus in each uterine horn) and unilateral (both fetuses in the same uterine horn, right or left). The risk of pregnancy loss during the first trimester of gestation for cows carrying twins may be from three to seven times higher than for cows carrying singletons, and from five to nine times higher for unilateral than for bilateral twins [7]. The risk of twin pregnancy is much more common in older cows [8,9] and so is the associated risk of abortion [7,10]. Twin pregnancies may account for 25% of all pregnancies on Day 90 of gestation in cows in their third lactation or more [11]. Thus, these cows carrying twins or singletons are a good model to investigate incidence patterns of twin pregnancies. Accordingly, the objectives of the present study were to: (a) gain information regarding the relative impact of twins in comparison with singletons in pregnant cows in their third lactation or more, (b) assess and expand information on the relative incidence of bilateral versus unilateral multiple pregnancies, (c) confirm corresponding laterality between embryos and corpora lutea, (d) tabulate the frequency of triplets and quadruplets, and (e) evaluate possible effects of environmental heat stress conditions on the incidence of multiple pregnancies. Due to the extensively described negative impact of twin births on reproductive performance in dairy herds, knowledge of incidence patterns of twin or multiple pregnancies is essential to design adequate management strategies for animals at risk.

## 2. Materials and Methods

### 2.1. Cattle, Herd Management, and Pregnancy Diagnosis

The study population was a commercial dairy herd of Holstein-Friesian lactating dairy cows in North-eastern Spain (latitude 41.13 N, longitude 0.24 E). During the study period (August 2014 to July 2018), the mean number of lactating cows in the herd was 2420 and mean annual milk production was 12230 kg per cow. Cows were milked three times daily and fed complete rations. Only healthy cows in their third lactation or more experiencing their first postpartum pregnancy after a spontaneous estrus and with complete information from insemination to pregnancy loss or parturition were included in the study. Thus, cows were included only once per lactation in the experiment. This protocol was designed to minimize variation related to a possible effect of a previous hormone treatment protocol for timed artificial insemination (AI) on the risk of multiple pregnancies [12]. All cows were artificially inseminated and the herd was maintained on a weekly reproductive health program, as described previously [11]. As a reference, pregnancy was recorded in 9414 out of a total of 22,848 (41.2%) inseminations during the study period. The calving rate for the total cows carrying singletons was 92% (7101/7719).

Pregnancy was diagnosed by transrectal ultrasonography from Days 28 to 34 post-AI using a portable B-mode ultrasound scanner equipped with a 5–10 MHz transducer (E.I. Medical IBEX LITE, E.I. Medical Imaging, Loveland, CO, USA). Each ovary was scanned in several planes by moving the transducer along its surface to identify luteal structures, and the number and location of corpora lutea (CL) were recorded. Scanning was then performed along the dorso/lateral surface of each uterine horn. The presence of twins was established through the observation of two embryos in different positions within one uterine horn on two screen scans or two embryos simultaneously present on the screen (unilateral twin pregnancy), or one embryo in each uterine horn (bilateral twin pregnancy). Triplets and quadruplets were also recorded.

The final study population comprised 4290 cows in their third lactation or more carrying twins, triplets, quadruplets (*n* = 1130), or singletons (*n* = 3160) during the study period. All cows with multiple pregnancies had at least one live embryo and, to favor pregnancy maintenance, received a GnRH dose at pregnancy diagnosis [11]. The viability of an embryo/fetus was confirmed by observation of a heartbeat in all exams.

Targeting to describing natural conditions, only unilateral multiple pregnancies were monitored until pregnancy loss or calving. Bilateral multiple pregnancies were excluded as twin reduction by amnion rupture [7], which is routinely performed in all bilateral pregnancies in this herd. The number of live fetuses was checked by ultrasound 58 to 64 days post-AI. Pregnancy loss was recorded when this exam proved negative. Multiple pregnancy reduction was recorded at this time when remnants of dead embryos were observed besides a live fetus. Cows showing abortion signs before Day 260 of gestation were recorded as aborting cows [13]. Abortion was, furthermore, confirmed in cows detected in estrus by a pedometer system and the date of abortion was recorded as 20 days before estrus. Only cows testing seronegative for *Neospora caninum* were included in the study, 18 *Neospora*-seropositive cows were removed. This is because, in previous studies on the same herd, the abortion rate for *Neospora*-seronegative cows carrying singletons during the second and third trimester of gestation was close to 0%, whereas 30% of their seropositive peers aborted [14]. All gynecological examinations and pregnancy diagnoses were performed by the first author.

### 2.2. Data Collection and Statistical Analysis

Since this study focused on twin pregnancies, data collection for each cow carrying singletons were reduced to the AI date, year (four consecutive years during the study period), lactation number, and conception 28 to 34 days post-AI. Additional data for twin pregnancies were number and location (right or left side) of embryos and corpora lutea, and presence of a dead embryo at pregnancy diagnosis. Pregnancy loss, embryo reduction, abortion, and parturition were also recorded in unilateral multiple pregnancies. Insemination dates were used to assess the effects of the time of the year (warm vs. cool period) on the incidence of twins. It should be noted that, in our geographical region, there are only two clearly differentiated weather periods: warm (May to September) and cool (October to April) [15,16]. Since triplet and quadruplet pregnancies did not influence a significance of differences between percentages of twin pregnancies, for analyses, triplets or quadruplets were grouped with bilateral twins when at least one embryo was located in each uterine horn or with unilateral twins when all embryos were located in the same uterine horn.

Possible significant differences between percentages were assessed using the Chi-square test. The level of significance was set at *p* < 0.05. Values are expressed as the mean ± standard deviation (S.D.).

## 3. Results

In response to 12,381 inseminations performed from 52-days to 152-days post-partum, 4290 cows became pregnant (34.6%): 3160 (73.7%) were singleton pregnancies and 1130 (26.3%) were multiple pregnancies of which 1117 (98.8%) were twins, 12 (1.1%) triplets, and one quadruplet (0.08%). The mean number of lactations at pregnancy diagnosis was 3.5 ± 0.6 (3–9 lactations): 3.6 ± 0.8 lactations (3–9 lactations) or 3.5 ± 0.7 lactations (3–8 lactations) for cows carrying singletons or multiple embryos, respectively. Since there were no differences in terms of years for the different parameters investigated, data were grouped as a single year (Table 1). The conception rate was significantly lower (*p* < 0.0001) for the warm (31.1%) than cool (37.5%) period, whereas multiple pregnancies relative to the total number of pregnancies (26.3%) were similar for both periods. Unilateral multiple pregnancies (54.4%) were significantly more frequent (*p* < 0.0001) than bilateral multiple pregnancies (45.6%). This difference is more pronounced during the warm period (58.4% versus 41.6%, *p* = 0.03). Unilateral multiple pregnancies in the left uterine horn were significantly (*p* < 0.0001) less frequent (40.5%) than those in the right uterine horn (59.5%) with similar proportions recorded in the warm and cool periods. The presence of a spontaneous dead co-twin was detected in 72 (11.7%) unilateral and 79 (15.3%) bilateral twin pregnancies, and these proportions were similar in multiple pregnancies for the warm (13.4%) and cool (13.3%) periods. Additional CL (more CL than embryos) were recorded in 15 cows carrying twins: nine in unilateral and six in bilateral twin pregnancies.

In five cows (0.16%) carrying singletons, the embryo was located on the side contralateral to its corresponding CL. All embryos in multiple pregnancies were located ipsilateral to their corresponding CL except four cows bearing unilateral twins. Two cows carrying twins in the right uterine horn had only one ipsilateral CL, whereas two further cows carrying unilateral twins including one in the right and the other in the left uterine horn, which had one ipsilateral plus one contralateral CL. These four cows delivered twins.

There were no differences among years for the different parameters investigated in unilateral multiple pregnancies and data were grouped as a single year (Table 2). The presence of a dead co-twin (11.7%) was recorded in similar proportions in the warm and cool periods. Embryo reduction was significantly lower and pregnancy loss was higher for the warm period than the cool period (6.7% versus 24.6%, and 33.8% versus 9%, respectively, *p* < 0.0001). Pregnancy loss in cows carrying a dead co-twin was higher in the warm period (73.3%) than the cool (9.5%) period (*p* < 0.0001). The abortion rate diagnosed before 260 days of gestation in cows maintaining gestation 58–64 days post-AI was 0% for cows experiencing embryo reduction and 42.6% for cows carrying twins with similar values observed in the warm and cool period. Abortion signs were recorded in 128 (77.1%) cows, whereas post-abortion estrus was observed in the remaining 38 (22.9%) cows in which abortion was confirmed following estrous signs. The average time to abortion was 176 ± 35 days and ranged from 122 to 251 days. Of the total of 615 unilateral multiple pregnancies, 53.2% continued to term when 36.4% twins and 16.7% singletons were delivered. The incidence of twins was similar for the warm and cool periods. Parturition rates in cows delivering singletons and in total cows carrying unilateral twins at pregnancy diagnosis were significantly lower (*p* < 0.0001) for the warm (6.7% and 40.9%, respectively) than for the cool periods (24.6% and 62.7%, respectively).

All five cows carrying unilateral triplets suffered pregnancy loss before 58–64 days of gestation, whereas all nine cows with additional CL remained pregnant at this time point.

## 4. Discussion

Our objective was to examine incidence patterns of multiple pregnancies in a population of old dairy cows (in their third lactation or more) carrying singletons or multiple embryos in their first postpartum pregnancy. These animals show a higher likelihood of multiple pregnancies compared to younger cows [8,9]. Furthermore, we only included pregnant cows following a spontaneous estrus, excluding those that had received hormone treatment for estrous synchronization and timed AI, as this may also increase the chances of multiple pregnancies [12]. Finally, cows were included only once per lactation in the experiment to avoid the possibility of a repeat twin pregnancy. The rationale for this is that a given cow will show significant repeatability of multiple ovulations [9,17]. Under these working conditions and despite a known negative effect of warm temperatures on conception rates, similar impacts of multiple pregnancies were observed during the warm and cool periods. In contrast, the unilateral/bilateral multiple pregnancy ratio varied from 1.07 (346/323) during the cool period to 1.4 (269/192) during the warm period. In other words, the incidence of unilateral multiple pregnancies increased significantly (*p* = 0.03) during the warm period compared to the cool one, and, to a similar extent, bilateral multiple pregnancies decreased from the cool period to the warm period by a factor of about 7%. However, the occurrence of triplet and quadruplet pregnancies was very low to be tabulated. More cows would be required to accurately assess this incidence in dairy cattle.

Most multiple pregnancies in cattle result from multiple ovulations, involving the simultaneous formation of two or more ovulatory follicles either from one or both ovaries [18,19]. The findings of our study suggest that the development of two pre-ovulatory follicles, one in each ovary, is impaired under heat stress conditions to a greater extent than when there are two co-dominant follicles in the same ovary. An easier interpretation is that, under heat stress conditions, two ovulatory follicles in one ovary are both more likely to ovulate than one follicle in each ovary. Ovarian follicular cooling is a prerequisite for ovulation in mammals [20,21,22], and local distribution of capillary blood-flow [23] and cooling in the same ovary [21,22] could determine whether an ovary would contribute to develop two dominant follicles. Specific ovarian tissues seem to require a lower temperature than other organs to maintain their functions [24] and follicular cooling is very sensitive to heat stress [25]. Warm conditions likely impair the potential for ovulation of one follicle in cows with bilateral co-dominant follicles. The observation that heat stress reduces the size of the dominant follicle [26,27,28] reinforces this idea. Besides this follicular size decrease, an increased ratio of antral fluid volume to that granulosa cell mass as the follicle expands would facilitate the generation of intra-ovarian temperature gradients [22]. Therefore, it is reasonable to propose that cooling of one follicle would favor cooling of its neighboring follicle in the same ovary under heat stress conditions. Further work is needed to confirm this rationale.

Several studies have shown that the right ovary and uterine horn in the cow are larger and more active than their left counterparts [29,30,31]. Here, a right-left unilateral multiple pregnancy ratio of 1.5 (366/249) was maintained throughout the years and across the warm and cool periods, which is consistent with the results of a previous extensive study [12]. This may explain the larger size of the right uterine horn and that asymmetries in genital tract morphology and physiology are secondary to gonad asymmetry. In addition, the fact that, following fertilization, the embryo passes into the uterine horn ipsilateral to the ovulatory ovary is extensively described in the cow. The presence of an embryo in the uterine horn contralateral to the corpus luteum is rare with reported rates of 0% (none in 643) [32], 0.1% (1 in 1000) [30], and 0.2% (7 in 3094) [33] of pregnancies. In the present study, just five embryos (0.16%) were found in the horn contralateral to their corresponding CL in single pregnancies. Among the multiple pregnancies, four unilateral twins had a single CL ipsilateral to the embryo in which two of them have a further contralateral CL. Likely, these embryos were monozygotic twins. Although the rate of monozygotic twinning over the total number of twin births has been reported as low in dairy cattle, its incidence of 5.5% [34] and up to 13.6% estimated among all twin births in cows in their third lactation or more [35] is of clear interest at the herd level. Monozygotic twins with a single ipsilateral CL are often diagnosed as single pregnancies [7].

The presence of a spontaneous dead co-twin at the time of pregnancy diagnosis is highly related to subsequent pregnancy loss, which occurs in more than 60% of cases [36,37]. Although its incidence was low in the present study (11.7%), 73% of cows carrying a dead co-twin suffered pregnancy loss before 58–64 days post-AI during the warm period, whereas only 12% losses were recorded during the cool period. It is likely that the beneficial effects of GnRH treatment at pregnancy diagnosis on twin pregnancies [11] favored pregnancy maintenance in these cows during the cool period. This effectiveness of GnRH treatment following the death of a co-twin may be explained by reduced pro-inflammatory and luteolytic responses to the debris of the dead embryo [7,11]. Fused embryonic circulations already present during the late embryonic period in twin pregnant cows [18,38] is likely the cause for this high risk of pregnancy failure during the warm period. However, remaining cows stay pregnant as single pregnancies with an additional CL, which is a determining factor for pregnancy maintenance [39]. Consistently, although only nine cows carrying twins with additional CL were monitored here, all maintained their pregnancies. The practice of embryo reduction also offers an additional CL during the early fetal period, and a particularly high incidence of this was observed during the cool period favored by the dead co-twin maintaining pregnancy. In an extensive recent study, it was confirmed that GnRH treatment at pregnancy diagnosis in twin pregnancies substantially reduces the likelihood of pregnancy loss attributable to an increased rate of twin reduction [11].

Of the 390 cows with twins 58–64 days post-AI (615 unilateral multiple pregnancies excluding 103 cows undergoing embryo reduction plus 122 suffering pregnancy loss), 166 (42.6%) suffered abortion before 260 days of gestation with similar values noted for the warm and cool season. The average time to abortion was 176 ± 35 days and ranged from 122 to 251 days. Following a considerable peak of losses (36.1%) before 60 days of gestation, which is when placentation highly sensitive to any kind of stress [2,3,4] finishes in the cow [40,41], the first abortion was registered on day 122 of gestation, averaging in the middle of the gestation period. These results are similar to those reported in a recent study on 672 unilateral twin pregnancies in which primiparous and secundiparous cows were also included [10]. In this case, mechanical stress arising from a lack of space for unilateral twins could perhaps explain why the risk of abortion was higher in the middle than at the end of the gestation period. Abortion was recorded in only 7 (1.3%) of 522 bilateral twin pregnancies [10].

## 5. Conclusions

First of all, it should be clarified that, despite our use of the general term multiple pregnancies given the low numbers of triplets and quadruplets detected in the participating cows, only twin pregnancies should be taken into consideration, as previously reported [8].

Irrespective of the lower conception rate during the warm period, similar proportions of multiple pregnancies were observed during the warm and cool period. However, the incidence of unilateral multiple pregnancies was higher during the warm period than the cool period, and the incidence of bilateral multiple pregnancies decreased in similar measure from the cool to the warm period. This observation warrants further investigation.

The presence of a spontaneous dead co-twin at pregnancy diagnosis was the main factor promoting both pregnancy loss during the warm period and embryo reduction during the cool period. Of the 269 cows with unilateral multiple pregnancies inseminated during the warm period, only 110 (40.9%) completed their gestation to term. This figure most likely exceeds the breakeven point to maintain this type of pregnancy in a dairy herd. Interest (or absence of interest) of twin reduction has been discussed in a recent commentary [42]. To improve herd economy, induced twin reduction is the best management option and the use of a luteolytic agent to induce abortion upon a diagnosis of twins that may be a further suitable option [6], but, more effectively, would be to prevent twin pregnancies via transfer of a single embryo or drainage of subordinate follicles at insemination [43,44]. Lastly, although twin births are lowly heritable in dairy cattle [9,35,45], artificial insemination centers could play a key role by considering twin pregnancy as a genetic trait in sire-selection programs.

## Figures and Tables

**Table 1:** Conception and multiple gestation patterns related to the presence of unilateral embryos (at least two embryos in the right or left uterine horn) or bilateral embryos (at least one embryo in each uterine horn) during the warm and cool periods 28–34 days post-artificial insemination.

Parameters	Warm Period (May–September)	Cool Period (October–April)	Total
Inseminations	5595	6786	12,381
Conception rate *	1742 (31.1%) ^a^	2548 (37.5%) ^b^	4290 (34.6%)
Multiple pregnancy **	461 (26.5%)	669 (26.3%)	1130 (26.3%)
Unilaterality ***	269 (58.4%) ^c^	346 (51.7%) ^d^	615 (54.4%) ^g^
Left uterine horn ****	111 (41.3%)	138 (39.9%)	249 (40.5) ^i^
Triplet	0	1	1
Right uterine horn ****	158 (58.7%)	208 (60.1%)	366 (59.5%) ^j^
Triplet	1	3	4
Bilaterality ***	192 (41.6%) ^e^	323 (48.3%) ^f^	515 (45.6%) ^h^
Triplet	1	6	7
Quadruplet	0	1	1
Presence of a spontaneous dead co-twin ***	62 (13.4%)	89 (13.3%)	151 (13.4%)
Additional CL (more CL than embryos) ***	8 (1.7%)	7 (1.0%)	15 (1.3%)

* Values with different superscript differ within rows or columns according the Chi-square test (*p* < 0.05): a–b, g–h, i–j (*p* < 0.0001), c–d, e–f (*p* = 0.03). * Percentage of pregnancies relative to inseminations. ** Percentages relative to total pregnancies. *** Percentages relative to multiple pregnancies including five unilateral triplets (unilaterality) and seven bilateral triplets plus one bilateral quadruplet (bilaterality). **** Percentages relative to unilateral multiple pregnancies.

**Table 2:** Reproductive performance of unilateral multiple gestations during the warm and cool periods.

Parameters	Inseminations During the Warm Period (May–September)	Inseminations During the Cool Period (October–April)	Total Pregnancies
Unilateral multiple pregnancies	269	346	615
Presence of a dead co-twin	30 (11.6%)	42 (12.1%)	72 (11.7%)
Embryo reduction *	18 (6.7%) ^a^	85 (24.6%) ^b^	103 (16.7%)
Pregnancy loss *	91 (33.8%) ^a^	31 (9%) ^b^	122 (19.8%)
Pregnancy loss in cows with a spontaneous dead co-twin **	22 (73.3%) ^a^	4 (9.5%) ^b^	26 (36.1%)
Abortion ***	68 (42.5%)	98 (42.6%)	166 (42.6%)
Type of parturition *			
Single	18 (6.7%) ^a^	85 (24.6%) ^b^	103 (16.7%)
Twins	92 (34.2%)	132 (38.2%)	224 (36.4%)
Total parturitions	110 (40.9%) ^a^	217 (62.7%) ^b^	327 (53.2%)

* Values with different superscript differ within rows according to the Chi-square test (a–b: *p* < 0.0001). * Diagnosed 58–64 days post-AI (percentages relative to total number of unilateral multiple pregnancies). ** Percentages relative to cows with a dead co-twin at pregnancy diagnosis (28–34 d post-AI). *** Diagnosed before 260 days of gestation (percentages relative to cows carrying twins 58–64 days post-AI).
